# Combined Action of Shiga Toxin Type 2 and Subtilase Cytotoxin in the Pathogenesis of Hemolytic Uremic Syndrome

**DOI:** 10.3390/toxins13080536

**Published:** 2021-07-29

**Authors:** Romina S. Álvarez, Fernando D. Gómez, Elsa Zotta, Adrienne W. Paton, James C. Paton, Cristina Ibarra, Flavia Sacerdoti, María M. Amaral

**Affiliations:** 1 Laboratorio de Fisiopatogenia, Departamento de Fisiología, Instituto de Fisiología y Biofísica Bernardo Houssay (IFIBIO Houssay-CONICET), Facultad de Medicina, Universidad de Buenos Aires, Buenos Aires 1121, Argentina; alvarez.s.romina@gmail.com (R.S.Á.); gomezfernandod@gmail.com (F.D.G.); ezotta@fmed.uba.ar (E.Z.); ibarra@fmed.uba.ar (C.I.); fsacerdoti@fmed.uba.ar (F.S.); 2 Cátedra de Fisiopatología, Facultad de Farmacia y Bioquímica, Universidad de Buenos Aires, Buenos Aires 1113, Argentina; 3 Research Centre for Infectious Diseases, Department of Molecular and Biomedical Science, University of Adelaide, Adelaide 5005, Australia; adrienne.paton@adelaide.edu.au (A.W.P.); james.paton@adelaide.edu.au (J.C.P.)

**Keywords:** Shiga toxin-producing *Escherichia coli*, hemolytic uremic syndrome, Shiga toxin type 2, Subtilase cytotoxin, co-action

## Abstract

Shiga toxin-producing *E. coli* (STEC) produces Stx1 and/or Stx2, and Subtilase cytotoxin (SubAB). Since these toxins may be present simultaneously during STEC infections, the purpose of this work was to study the co-action of Stx2 and SubAB. Stx2 + SubAB was assayed in vitro on monocultures and cocultures of human glomerular endothelial cells (HGEC) with a human proximal tubular epithelial cell line (HK-2) and in vivo in mice after weaning. The effects in vitro of both toxins, co-incubated and individually, were similar, showing that Stx2 and SubAB contribute similarly to renal cell damage. However, in vivo, co-injection of toxins lethal doses reduced the survival time of mice by 24 h and mice also suffered a strong decrease in the body weight associated with a lowered food intake. Co-injected mice also exhibited more severe histological renal alterations and a worsening in renal function that was not as evident in mice treated with each toxin separately. Furthermore, co-treatment induced numerous erythrocyte morphological alterations and an increase of free hemoglobin. This work shows, for the first time, the in vivo effects of Stx2 and SubAB acting together and provides valuable information about their contribution to the damage caused in STEC infections.

## 1. Introduction

Shiga-toxigenic *Escherichia coli* (STEC) is a food-borne pathogen responsible for different clinical conditions, including bloody diarrhea, hemorrhagic colitis, and hemolytic uremic syndrome (HUS) [1]. STEC is present in the gut of several animal species, although ruminants and especially cattle are considered the main reservoir of these bacteria [2]. Serotype O157:H7 is the most prevalent etiological agent of this pathology in the world, but many other non-O157:H7 serotypes have also been isolated from patients with HUS [3].

HUS is clinically characterized by microangiopathic hemolytic anemia, thrombocytopenia, and variable degrees of kidney injury [4]. Argentina has the highest worldwide incidence of HUS caused by STEC infections. Despite the fact that the origin of this epidemiological situation has not been clearly established so far, the most widely accepted hypothesis is the coexistence of several factors including food contamination, person-to-person contact, poor hygiene practices, and the circulation of more virulent strains, among others [5]. From 2014 to 2018, a median of 314 new cases per year were notified by the National Health Surveillance System and the annual incidence was 6.52 cases per 100,000 children under five years of age [6]. In Argentina, HUS associated with STEC infections is the principal cause of acute renal injury in pediatric age groups and the second most recurrent cause of chronic renal disease [1,7].

The severity of HUS ranges from a mild clinical condition to a severe and fulminant disease affecting multiple organs such as the gut, kidneys, heart, lungs, pancreas, and central nervous system [8].

STEC produces different virulence factors such as Shiga toxin type 1 (Stx1) and/or type 2 (Stx2) and their variants [9]. To date, only four variants of Stx1 have been identified: Stx1a, Stx1c, Stx1d, and Sx1e which rarely cause disease in humans, and when they do, they produce mild disease [10,11,12]. Regarding Stx2, there are several subtypes: Stx2a, Stx2b, Stx2c, Stx2d, Stx2e, Stx2f, Stx2g, Stx2h, Stx2i, Stx2j, Stx2k, and Stx2l with different pathogenic potential [13,14]. Strains that express Stx2 are frequently associated with the most serious cases of HUS [15] with Stx2a thought to cause more severe disease than Stx2c [16].

Furthermore, some Locus for Enterocyte Effacement (LEE)-negative STEC not only produce Stx2 but also Subtilase cytotoxin (SubAB), which may contribute to the pathogenesis of HUS [17]. Two variants of SubAB were described, SubAB_1_ and SubAB_2_ types, and their genes were located on plasmids or the chromosome, respectively. SubAB_1_ was the first reported and was encoded on the virulence plasmid pO113 of an O113:H21 STEC strain [17]. In addition, SubAB_2_ was classified into three subtypes: SubAB_2-1_, encoded on the pathogenicity island (PAI) SE-PAI [18,19], and its allelic variant SubAB_2-2_ encoded on an outer membrane efflux protein locus [20]. The third allelic variant has been designated SubAB_2-3_ and was associated with a gene predicted to encode a protein of yet-unknown function, located upstream of the SubAB locus [21].

Stx and SubAB belong to the AB_5_ cytotoxin family and are composed of a catalytic A subunit and a pentamer of receptor recognition domain B subunits [17,22]. Stx binds to the glycolipid receptor globotriaosylceramide (Gb3) on the target cells [23] and Stx-receptor complexes are internalized by endocytosis and transported via the Golgi Apparatus to the endoplasmic reticulum (ER). It is there the A-subunit is cleaved into A1 and A2 fragments releasing the active A1-fragment, which is retrotranslocated into the cytosol where inactivates ribosomes. This results in inhibition of protein synthesis and together with ribotoxic stress responses, leads to apoptosis. The transient unfolded form of the toxin triggers a cell stress response pathway that induces cytokine production, autophagy, and apoptosis. Thus, cell death occurs by multiple pathways, including ER stress, apoptosis, and autophagy [24].

The B subunit pentamer of SubAB recognizes cell surface glycoprotein receptors displaying sialic acid (particularly *N*-glycolylneuraminic acid) moieties [25]. This triggers uptake of the holotoxin, which undergoes retrograde transport to the ER where the A subunit, a highly specific subtilase-family serine protease, cleaves the ER chaperone protein BiP/Grp_787_ [26], resulting in activation of ER stress-sensor proteins [27]. The induction of this signaling produces a multiplicity of cell responses such as inhibition of protein synthesis, cell cycle arrest, apoptosis, inhibition of iNOS synthesis, stress granule formation, and autophagy [28,29,30,31,32].

To date, SubAB has not been detected in patients, although several STEC serotypes expressing SubAB have been associated with cases of HUS worldwide [33]. Our group and others have provided information suggesting that SubAB could be a relevant pathogenic factor in STEC infections since it may contribute to HUS pathophysiology. Supporting this idea, we have previously demonstrated the in vitro deleterious effects of SubAB on human renal cells. We were able to show that SubAB can cause endothelial and epithelial damage with similar characteristics to the damage seen in HUS pathogenesis [34,35,36,37]. Other authors also showed in vivo that the inoculation of purified SubAB in mice and rats induced the appearance of HUS symptoms [38,39]. In this sense, it was suggested that SubAB is able to enhance the clinical manifestations of STEC infection [40].

The presence of SubAB genes and the production of this cytotoxin has been described in two Stx-negative *E. coli* strains associated with human diseases [41]. However, assessment of its contribution to HUS is complicated by the fact that most strains producing SubAB also express Stx1 or Stx2 [42]. In this sense, there is almost no information available about the effects in vitro or in vivo of Stx2 and SubAB together, as might occur during STEC infections. Interestingly, we recently showed that while Stx2 increased IL-8 release, Stx2 + SubAB had no influence or even reduced IL-8 production, indicating that SubAB could modify the inflammatory response caused by Stx2 [37]. In this sense, Wang et al. described similar results on the human macrophage U937 cell line [43].

Considering these previous studies and the strong possibility that Stx2 and SubAB may be present simultaneously during STEC infections, the aim of this work was to analyze the effect of these two toxins together in vitro on monocultures and cocultures of human glomerular endothelial cells (HGEC) and a human proximal tubular epithelial cell line (HK-2) as well as in vivo in mice after weaning.

## 2. Results

### 2.1. Co-Incubation (Stx2 + SubAB) Reduced In Vitro Cell Viability without Additive or Synergistic Effects

**Figure 1:**
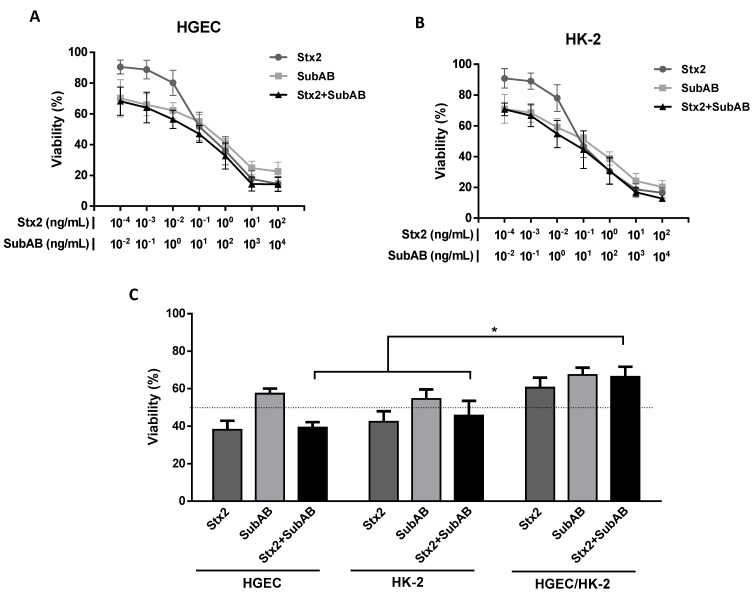
Reduction of cell viability on HGEC and HK-2 cells by the co-incubation (Stx2 + SubAB). HGEC (**A**) and HK-2 (**B**) cells were incubated with different concentrations of Stx2 (1 × 10^−4^ to 1 × 10^2^ ng/mL), SubAB (1 × 10^−2^ to 1 × 10^4^ ng/mL) or with both toxins together (Stx2 + SubAB) in growth-arrested conditions for 72 h. Then, cell viability was analyzed by neutral red uptake. Absorbance in each well was read at 540 nm. One hundred percent represents cells incubated under identical conditions but without toxin treatment (Control). Results are expressed as means ± SEM, (*n* = 8). (**C**) HGEC and HK-2 monocultures and HGEC/HK-2 cocultures were exposed to the 50% cytotoxic dose (CD50) for Stx2 (0.1 ng/mL), SubAB (10 ng/mL) or Stx2 + SubAB (0.1 ng/mL + 10 ng/mL) for 72 h. Then, cell viability was analyzed by neutral red uptake. Results are expressed as the means ± SEM, (*n* = 9), * *p* < 0.05.

After 72 h of incubation with either Stx2, SubAB, or Stx2 + SubAB there was a significant decrease in the viability of HGEC and HK-2 cells relative to controls in a dose-dependent manner. Differences between co-incubation and Stx2 or SubAB alone were not statistically significant. In Figure 1A,B it is possible to observe that at the lowest toxin concentrations, co-incubation caused a similar decrease in the cell viability to that seen with incubation with SubAB alone. The decrease in cell viability caused by co-incubation with Stx2 + SubAB showed a tendency to be higher than that caused by Stx2 treatment alone, although this difference did not reach statistical significance. For the highest toxin concentrations, Stx2, SubAB, and Stx2 + SubAB showed similar cytotoxic effects.

The co-incubation of Stx2 and SubAB CD50 significantly decreased the cell viability of cocultures relative to controls, but this effect was not significantly different from that caused by each toxin individually. In addition, co-incubation cytotoxicity was attenuated by approximately 20% on cocultures compared to HGEC and HK-2 monocultures (Figure 1C).

### 2.2. Stx2 and SubAB Minimum Lethal Dose

**Figure 2:**
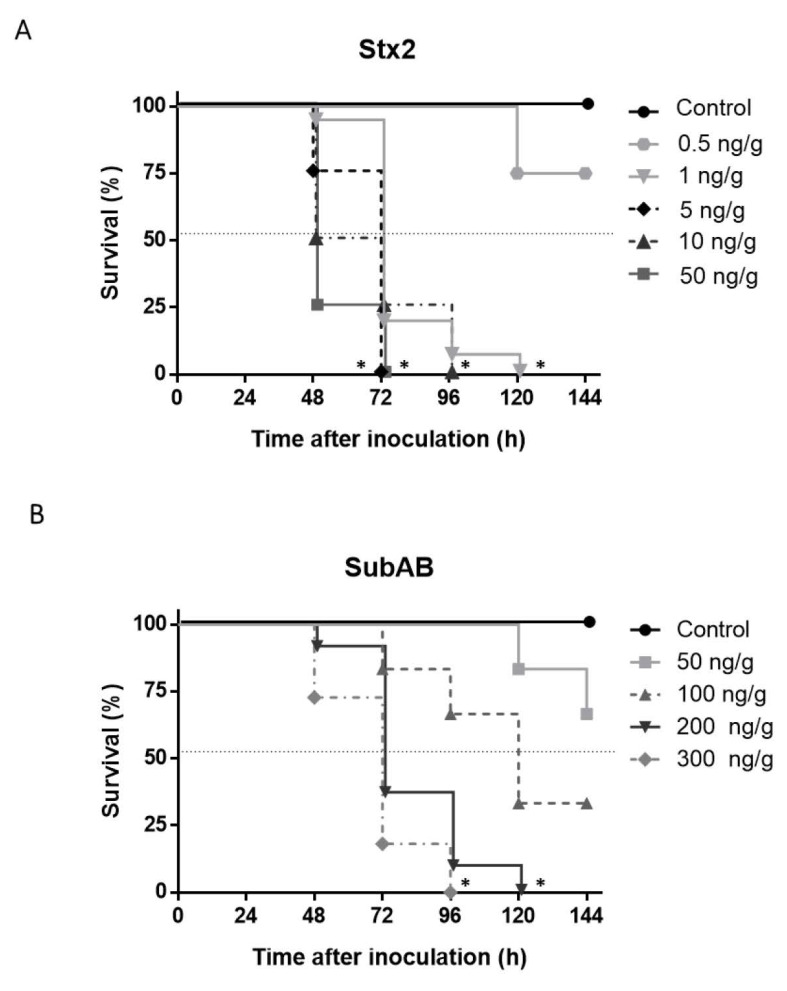
Stx2 and SubAB minimum 100% lethal dose. Mice were injected i.p. with different doses of (**A**) Stx2 (0.5, 1, 5, 10, 50 ng/g bwt) and (**B**) SubAB (50, 100, 200, 300 ng/g bwt). Control mice received the same volume of PBS. Survival of mice was daily monitored after toxins inoculation. (*n* = 8), Log-rank test, * *p* < 0.0001.

The minimum 100% lethal dose for each toxin was established by mouse survival evaluation after Stx2 or SubAB injection. As shown in Figure 2, 100% of death was obtained from 72 h to 120 h after injection of an Stx2 dose of 1 ng/g bwt and a SubAB dose of 200 ng/g bwt. These doses were used in the rest of the experiments.

### 2.3. The Co-Treatment Decreased the Survival Time and the Body Weight in Mice

**Figure 3:**
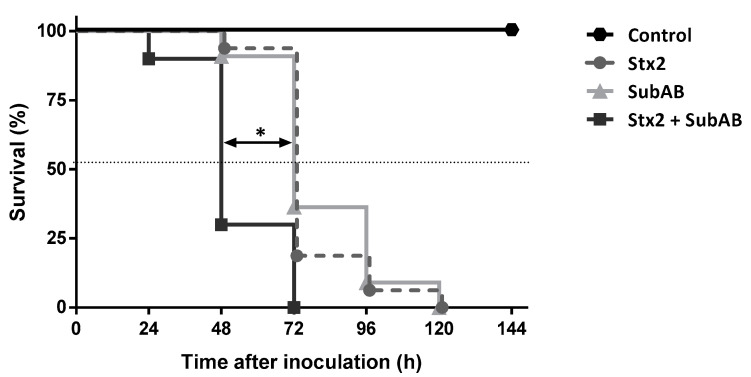
Effects of co-treatment (Stx2 + SubAB) on mice survival. Mice were inoculated i.p. with a lethal dose of Stx2 (1 ng/g bwt), SubAB (200 ng/g bwt) or Stx2 + SubAB (1 ng/g bwt + 200 ng/g bwt). Control mice received the same volume of PBS. Survival of mice was monitored daily after toxins inoculation. Log-rank test corresponding to at least four experiments, Stx2 + SubAB vs. Stx2 or SubAB, * *p* < 0.001 (*n* = 16).

**Figure 4:**
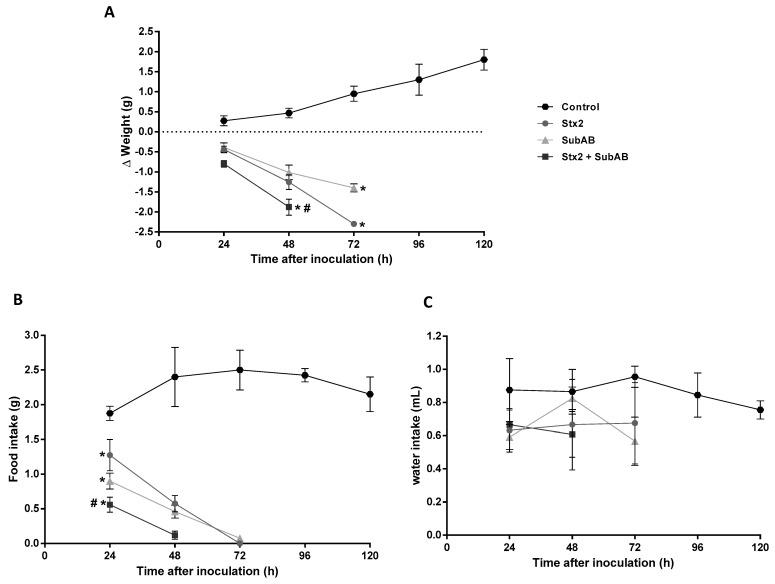
Evaluation of body weight, food, and water intake after co-treatment. Time course of body weight (**A**), food intake (**B**) and water intake (**C**) of mice inoculated i.p. with Stx2 (1 ng/g bwt), SubAB (200 ng/g bwt), Stx2 + SubAB ((1 + 200) ng/g bwt) or PBS (Control). Each point of the curve represents the means ± SEM, corresponding to at least two experiments, (*n* = 7). (**A**,**B**) Stx2, SubAB or Stx2 + SubAB vs. Control, * *p* < 0.05. Stx2 + SubAB vs. Stx2 or SubAB, # *p* < 0.05. (**C**): there were no significant differences between groups.

The in vivo administration of Stx2 + SubAB (co-treatment) caused significant differences compared to Stx2 or SubAB treatment. Figure 3 shows that most mice inoculated with Stx2 + SubAB died at 48 h after treatment while most mice injected with Stx2 or SubAB died at 72 h after treatment. In addition, while control mouse body weight increased every day after PBS treatment, mice from the groups treated with toxins suffered a significant decrease in the body weight (Figure 4A). This decrease was coincident with a reduction in food intake (Figure 4B). Moreover, 48 h after toxin injection, both body weight, and food intake decrease were significantly greater for the co-treatment than for each toxin individually (Figure 4A,B). No significant differences were obtained between groups for water intake (Figure 4C).

### 2.4. Renal Damage and Levels of Urea in Serum Are Increased by the Co-Treatment

Considering the kidneys are one of the major affected organs in HUS patients, we analyzed the renal histology and plasma levels of creatinine and urea of mice after 24 h of toxins injection.

**Figure 5:**
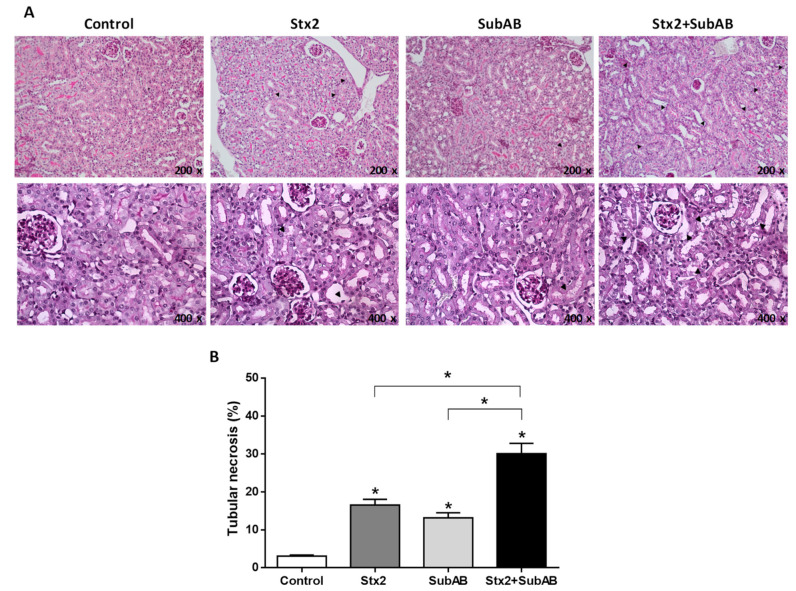
Kidney histological examination of co-treated mice. (**A**) Optical microscopy of kidneys paraffin sections obtained after 24 h post injection with Stx2, SubAB, Stx2 + SubAB or PBS (Control) and stained with PAS (200x and 400x). Tubular necrosis (black arrow). (**B**) Quantification of tubular necrosis by semi-quantitative scoring. Ten fields/kidney were quantified (*n* = 3). Data are expressed as means ± SEM of tubular necrosis percentage. Stx2, SubAB, Stx2 + SubAB vs. Control, or Stx2 + SubAB vs. Stx2 SubAB * *p* < 0.05.

As it is shown in Figure 5A, kidneys of mice treated with Stx2 or SubAB exhibited isolated foci of tubular necrosis with loss of the brush border and widening of the tubular lumen compared to controls. However, kidneys of mice injected with Stx2 + SubAB exhibited a more extensive tubular necrosis with loss of the brush border, flattening of tubular cells and widening of tubular lumen, breakage, and detachment of tubular cells from the basement membrane.

Quantification analysis (Figure 5B) showed that injection of Stx2 and SubAB caused a significant increase in tubular necrosis compared to controls. Furthermore, a significantly greater degree of tubular necrosis was found in kidneys of mice co-injected with Stx2 and SubAB compared to mice that received toxins separately.

**Figure 6:**
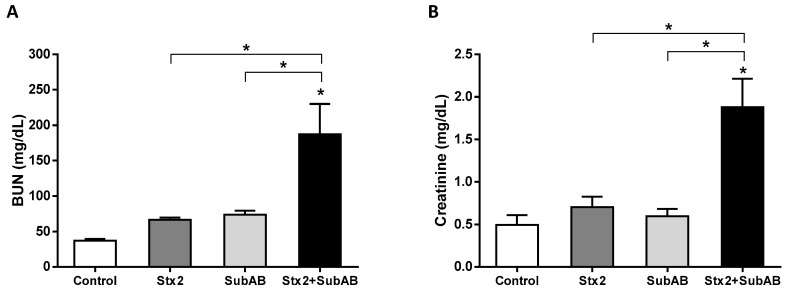
Effect of Stx2 + SubAB co-treatment on mice renal function. (**A**) BUN (blood urea nitrogen) (*n* = 6) and (**B**) creatinine (*n* = 3) levels in plasma after 24 h post injection with Stx2, SubAB, Stx2 + SubAB or PBS (Control). Results are expressed as means ± SEM, * *p* < 0.05; Stx2 + SubAB vs. Stx2 or SubAB.

In addition, renal function was analyzed by measuring plasma levels of creatinine and urea (BUN) after 24 h of inoculation (Figure 6). While mice treated with Stx2 or SubAB did not show any significant change in urea levels, Stx2 + SubAB caused a very significant increase compared to controls and mice injected with toxins individually.

### 2.5. Co-Treatment Increases Damaged Erythrocytes and Reticulocytes

**Figure 7:**
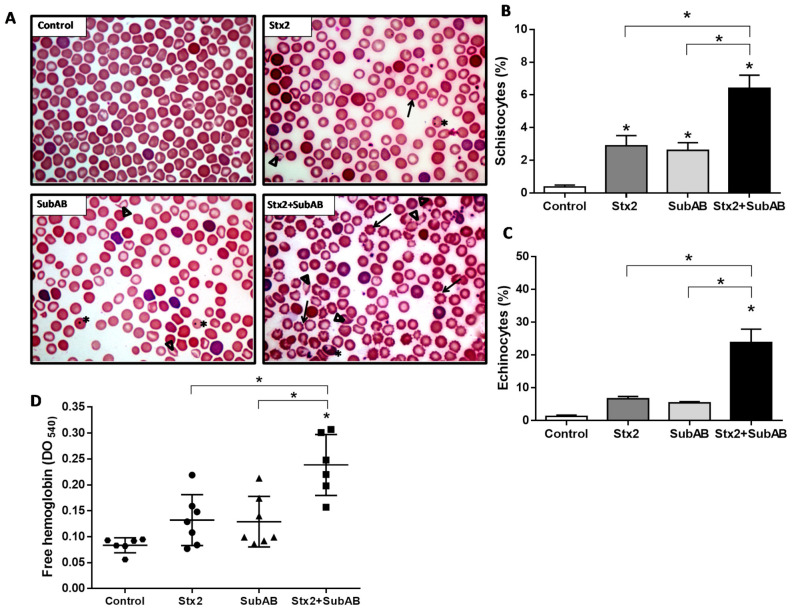
Erythrocytes morphology and levels of plasma free hemoglobin in co-treated mice. (**A**) Optical microscopy of peripheral blood smears obtained of mice after 24 h after injection with Stx2, SubAB, Stx2 + SubAB or PBS (Control) and stained with Wright–Giemsa, 1000x. Echinocytes (black arrow), Howell Jolly bodies (*), and Schistocytes (∆). (**B**) Schistocytes (%). (**C**) Echinocytes (%). Results of erythrocytes morphology alterations quantification are expressed as means ± SEM, (*n* = 4). Stx2 + SubAB vs. Control, Stx2 or SubAB, * *p* < 0.05. (**D**) Plasma free hemoglobin levels (OD_540_ nm). Data are expressed as means ± SD, (*n* = 6). Stx2 + SubAB vs. Control, Stx2 or SubAB, * *p* < 0.05.

We analyzed the morphology of mouse erythrocytes 24 h after injection with Stx2, SubAB, Stx2 + SubAB, or PBS (Control). As shown in Figure 7A, mice co-injected with Stx2 + SubAB exhibited schistocytes, echinocytes, Howell Jolly bodies, hypochromia, microcytosis, and an increase of reticulocytes. Mice singly inoculated with Stx2 or SubAB toxins showed some of these erythrocyte morphological alterations but to a lesser extent.

In addition, we found that Stx2 + SubAB induced a significantly higher percentage of schistocytes and echinocytes relative to Stx2 or SubAB treatments (Figure 7B and Figure 7C, respectively).

Finally, the presence of free hemoglobin in peripheral blood circulation was analyzed. Mice injected with Stx2 + SubAB exhibited a significant increase of free hemoglobin relative to controls and mice treated with the toxins administered separately (Figure 7D). These last groups showed only a (non-significant) trend towards increased free hemoglobin compared to controls.

## 3. Discussion

The etiology of HUS is multifactorial and involves complex interactions between bacterial and host factors [44]. In this sense, the initial bacterial inoculum, the type of Stx, and the presence of additional bacterial virulence factors, such as adhesins, proteases, and other toxins, as well as the particular characteristics of the host’s inflammatory and hemostatic response are variables that influence HUS evolution and severity [45].

Stx is the main STEC virulence factor involved in the pathogenesis of HUS. However, a particular group of STEC negative for LEE pathogenicity island also produce Subtilase cytotoxin [17]. In Argentina, as in the rest of the world, several *E. coli* strains producing Stx and SubAB have been identified [33,40,42]. Nevertheless, to date, SubAB has not been detected in the blood and tissues of patients with HUS. One reason is due to systematic studies in humans have not been carried out. Another reason is that free toxin is unlikely to remain in the circulation, as it would rapidly bind to target glycans. Detecting it in tissues is also problematic, as it would be internalized by target cells. Therefore, one of the most intriguing aspects of the pathophysiology of HUS is the possible contribution of Stx2 and SubAB together during STEC infections.

To examine this, we first analyzed in vitro the effects of co-treatment and found similar effects on the viability of HGEC and HK-2 cells (in monocultures and cocultures) as was seen for individual toxins. Consequently, at least in our in vitro models, both toxins together and separately appeared to contribute equally to renal cell damage. This result may be possible considering that Stx2 and SubAB recognize different receptors whose expression levels in HGEC and HK-2 can also be different. In this sense, we previously found that although HK-2 cells showed a higher concentration of Gb3 receptor than HGEC cells, both types of cells exhibited the same sensitivity to Stx2 [34,46]. However, as it was documented, the heterogeneity in Gb3 receptors between different cells could define the final susceptibility to Stx2 [47,48]. With respect to N-glycolylneuraminic acid, we have not identified yet the presence of these SubAB receptors on HGEC and HK-2 but other authors have reported SubAB receptors on HeLa cells [49] and HUVEC [50], used as a model of epithelial and endothelial cells, respectively.

We then examined co-injection of Stx2 and SubAB lethal doses in mice after weaning. Interestingly and unlike the in vitro results, we obtained evidence of more severe damage after Stx2 and SubAB co-injection than occurred when toxins were administrated separately. In this sense, co-injection reduced mouse survival time by 24 h and, in addition, mice suffered a stronger decrease in the body weight associated with diminished food intake and probably related to the deterioration in the general state, since piloerection and inactivity were evidenced. Bodyweight loss induced by both Stx2 and SubAB was also observed in several HUS models [17,51,52]. 

Furthermore, we found that in renal tissues both toxins together caused extended tubular necrosis in a higher percentage of compromised proximal tubules than that observed in mice individually inoculated with Stx2 or SubAB. According to this observation, tubular necrosis is a feature of human HUS, since in patients direct damage to the proximal tubules has been observed [53,54], as well as in the glomeruli and arterioles [55]. Additionally, plasma creatinine and urea levels were also found to be raised in co-injected mice, suggesting decreased glomerular filtration as an indicator of abnormal renal function. So, in our model, co-injection of Stx2 and SubAB lead to a clear worsening in renal function that was not so evident with either toxin individually. In accordance with our results, some authors have documented histological renal alterations and also an increase in the glomerular endothelial damage and blood urea and creatinine levels in mice after injection of purified Stx [51,52,56]. There is also evidence that the injection of SubAB in mice induces microangiopathic hemolytic anemia, thrombocytopenia, and renal failure, mimicking the HUS clinical triad caused by Stx in humans [38]. Furthermore, survival time decreased with the increase of SubAB dose. In mice inoculated with a dose of SubAB higher than that used in this work (5 µg–25 µg), massive microvascular thrombosis and histological injury in kidneys were found after 72 h of treatment. Elevated urea levels were also seen within 24 h as an indicator of renal failure [38]. Additionally, Seyahian et al., in a model of rats injected with a sublethal dose of SubAB observed microalbuminuria as a consequence of the disruption of the glomerular filtration barrier and alterations in the proximal tubule protein reabsorption mechanisms [39].

In our in vivo model, hematological studies demonstrated that co-treatment induced several erythrocyte morphological alterations and a compensatory increase in circulating reticulocytes. Erythrocytes from mice inoculated with Stx2 or SubAB individually only exhibited some of these morphological alterations.

The co-injected mice also exhibited the appearance of circulating echinocytes, usually observed in human cases of renal failure due to a high plasma concentration of certain metabolic derivatives, especially guanidine derivatives that can cause a hemolytic effect [57]. In addition, increased free hemoglobin was detected in co-injected mice, which suggests that co-treatment leads to greater intravascular hemolysis than when toxins are administered individually.

In this sense, Wang et al., also reported a high percentage of fragmented erythrocytes after 48 h of SubAB injection and the appearance of free hemoglobin after 72 h. In addition, the osmotic fragility of red blood cells was not affected by SubAB treatment, suggesting that intravascular hemolysis was caused by the presence of microangiopathy and not by a direct effect of SubAB on the red blood cell membrane [38]. So, the intravascular hemolysis could be a consequence of the mechanical damage suffered by the erythrocytes in microcirculation vessels, possibly obstructed by small thrombi formed due to endothelial activation and damage triggered by the joint action of Stx2 and SubAB. With respect to the effects of SubAB on human erythrocytes, no evidence is documented yet.

## 4. Conclusions

In summary, this work provides very valuable and interesting information on the in vivo effects of both toxins together, since up to now, neither experimental nor clinical evidence about the contribution of Stx2 and SubAB in STEC infections has been documented.

The combined action of Stx2 and SubAB can cause more serious kidney damage and the appearance of typical HUS features in humans such as partial or total deterioration of kidney function, erythrocyte alterations, increase of free hemoglobin, probably associated with microangiopathic hemolytic anemia [1,58], and even death. Further studies are necessary to delineate the role of SubAB in the pathogenesis of HUS. This information will allow a better understanding of the pathogenicity of STEC and contribute to the identification of new therapeutic targets.

## 5. Materials and Methods

### 5.1. Reagents

Purified Stx2a was provided by Phoenix Laboratory, Tufts Medical Center, Boston, MA, USA. SubAB was purified from recombinant *E. coli* by Ni-NTA chromatography via a His_6_ tag fused to the C-terminus of the B subunit, as described previously [17]. Purity was greater than 98%, as judged by SDS-PAGE and staining with Coomassie Blue.

### 5.2. Human Primary Glomerular Microvascular Endothelial Cell Culture

Human glomerular endothelial cells (HGEC) were isolated from kidney fragments removed from normal areas from different pediatric patients with segmental uropathies or tumors in one pole and normal creatinine that were undergoing nephrectomies performed at National Hospital “Alejandro Posadas”, Buenos Aires, Argentina (written informed consent was obtained from the next of kin, caretakers, or guardians on the behalf of the minors/children participants involved in our study, n°: 035 LUP1S0/19 (13)). The study was conducted in accordance with the Declaration of Helsinki, and The Ethics Committee of the University of Buenos Aires approved the use of human renal tissues for research purposes. Once isolated, HGEC were grown in M199 media supplemented with 20% fetal calf serum (FCS), 3.2 mM L-glutamine, 100 U/mL penicillin/streptomycin (GIBCO, Waltham, MA, USA), and 25 µg/mL endothelial cell growth supplement (ECGS, Sigma, St. Louis, MO, USA) as previously described [34]. Experiments were done with HGEC between 2–7 passages and previously characterized for positive expression of von Willebrand factor and platelet/endothelial cell adhesion molecule 1 (PECAM-1). In addition, experiments were performed at growth-arrested conditions using a medium with half FCS concentration (10%) and without ECGS [34].

### 5.3. Tubular Human Epithelial Cell Line Culture

The human proximal tubular epithelial cell line (HK-2) was acquired from American Type Culture Collection (ATCC, Manassas, VA, USA). Cells were grown in DMEM/F12 medium (Sigma Aldrich, St. Louis, MO, USA) with 10% FCS, 100 U/mL penicillin/streptomycin (GIBCO, Waltham, MA, USA), 2 mM L-glutamine (GIBCO, Waltham, MA, USA), 15 mM HEPES. Experiments were done at growth-arrested conditions using a medium without FCS.

### 5.4. Monocultures and Co-Cultures of Renal Endothelial and Epithelial Cells

HGEC and HK-2 co-cultures were done using Millicell cell culture inserts (PIHP01250, Millipore, Billerica, MA, USA). HGEC (5.10^4^) were seeded on the lower side of the Millicell filter (0.4 µm membrane pore size) and allowed to attach during 12–16 h. Then, inserts were inverted, and HK-2 (7.10^4^) was seeded into the upper side. Co-cultures were maintained under growth conditions in HGEC complete medium. To obtain HK-2 and HGEC monocultures, the same method was performed but partner cells were not seeded. The integrity of HGEC and HK-2 monolayers and HGEC/HK-2 bilayers was verified by using a Millicell-ERS electric resistance system (Millipore, Billerica, MA, USA) calibrated for each measurement, as previously described [36]. To analyze the effects of Stx2 and SubAB on monocultures and cocultures, Stx2 or SubAB or Stx2 + SubAB were incorporated into the bottom compartment (HGEC compartment).

### 5.5. Cell Viability Evaluation

The effect of co-incubation (Stx2 + SubAB) on the cell viability was evaluated by the neutral red cytotoxicity assay as previously reported [36]. HGEC and HK-2 cells were incubated or not during 72 h with Stx2 (1 × 10^−4^ to 1 × 10^2^ ng/mL), SubAB (1 × 10^−2^ to 1 × 10^4^ ng/mL) or both Stx2 + SubAB. For controls, only growth-arrested conditioned medium was employed. To compare the effect of co-incubation on HGEC and HK-2 monocultures and HGEC/HK-2 cocultures, cells were incubated or not for 72 h with the 50% cytotoxic dose (CD50) for Stx2 (0.1 ng/mL) or SubAB (10 ng/mL) or both Stx2 + SubAB. After 72 h of treatment, freshly diluted neutral red (10 µg/mL, Sigma Aldrich, St. Louis, MO, USA) was incorporated (10 µg/mL, final concentration). Then, cells were incubated for an additional 1 h at 37 °C in 5% CO_2_. Later, cells were washed and fixed with 200 µL of a solution with 1% CaCl_2_ + 1% formaldehyde and finally lysed with 200 µL of a solution with 1% acetic acid in 50% ethanol, to solubilize the neutral red. Absorbance was measured in each well at 540 nm by an automated plate spectrophotometer. Results were expressed as cell viability percentage and 100% represents cell viability of controls.

### 5.6. Animals

Immature male BALB/c mice were acquired immediately after weaning (17–21 days of age, approximately 8–11 g of body weight) from the Animal Facility of the School of Veterinary Sciences. The animals received food and water ad libitum and were housed under controlled conditions of light (12 h light; 12 h dark) and temperature (23–25 °C). This study was carried out in strict accordance with the recommendations detailed in the Guide for the Care and Use of Laboratory Animals of the National Institutes of Health. Protocols were approved by the Committee for the Care and Use of Laboratory Animals of the University of Buenos Aires (CICUAL, Permit Number 2438/2019). Four animals were used in each experimental group. The experiments were repeated at least three times.

### 5.7. Setting of Stx2 and SubAB Minimum Lethal Dose

In order to establish the minimum 100% lethal dose of Stx2 and SubAB after 72 h of treatment, mice were randomly divided into different groups. A single different dose of Stx2 (ng of Stx2/g of body weight: bwt) or SubAB (ng of SubAB/g bwt) was administered via intraperitoneal injection (i.p.). Control mice were injected with the same volume (1 µL/g bwt) of PBS. Administered doses for Stx2 were: 0.5, 1, 5, 10 and 50 ng/g bwt and for SubAB: 50, 100, 200 and 300 ng/g bwt. Survival was evaluated periodically and time to death registered.

### 5.8. Evaluation of Mouse Survival and General Status

BALB/c mice were inoculated i.p. with the established minimum lethal dose of Stx2 and SubAB after 72 h of treatment: 1 ng Stx2/g bwt, 200 ng SubAB/g bwt or (1 ng Stx2 + 200 ng SubAB)/g bwt. The Control group received PBS. Periodically, survival, body weight and ingestion of food and water were analyzed. Data of weight are presented as Δ weight (body weight at n day)−(body weight at 0 days) after injection. Mice were individually housed, weighed, and checked for water (mL) and food intake (g) every 24 h post injection. In addition, the general status of the mice was analyzed through the appearance of signs of disease such as piloerection and inactivity.

### 5.9. Blood Samples and Renal Tissue Collection

Some mice were anesthetized with 100 μg ketamine and 10 μg diazepam per g of body weight, intraperitoneally. For performing hematological studies, blood samples were obtained by intracardiac puncture in blood collection tubes containing EDTA. After that, mice were perfused first with a 0.9% NaCl solution (*w*/*v*) and then with 4% (*v*/*v*) formaldehyde in PBS. Finally, the kidneys were removed.

### 5.10. Renal Histoarchitecture

Renal tissues were fixed for 48 h with formalin 10% in PBS 0.1M (pH 7.4), dehydrated, and embedded in paraffin. Sections of 5 µm were made using a microtome (Leica RM 2125, Wetzlar, Germany) and mounted on 2% silane coated slides. The sections were stained with Periodic Acid-Schiff (PAS) and then observed by light microscopy (Nikon Eclipse 200, Melville, NY, USA). Every image was scored by two independent blinded and independent observers. Renal damage was evaluated by quantifying tubular necrosis by semi-quantitative scoring from images of the sections stained with PAS. The tubular necrosis was characterized by loss of the brush border, flattening of cells, rupture, and detachment of tubular cells from the basement membranes. The total percentage of damage for each treatment was calculated from the average of 10 fields per kidney. The results were expressed as the mean ± SEM of the percentage of tubular necrosis.

### 5.11. Renal Function

Urea and creatinine concentration (mg/dL) were determined in plasma at 24 h post injection by using commercial reagents (Kinetic Creatinine AA, Wiener Lab, Rosario, Argentina) (Urea color 2R, Wiener Lab, Argentina). For creatinine and blood urea nitrogen (BUN), absorbance was measured at 500 nm and 570 nm, respectively, by an automated plate spectrophotometer.

### 5.12. Hematological Studies

#### 5.12.1. Blood Smear

Blood smears were made by spreading a drop of blood on a slide. Then, they were allowed to dry at room temperature and fixed by adding methanol for 3 min. Subsequently, blood smears were stained with Wright–Giemsa for 4 min and finally visualized by light microscopy at 1000× to analyze the erythrocyte cell morphology.

#### 5.12.2. Erythrocyte Morphology

The presence of schistocytes, echinocytes, Howell Jolly bodies, hypochromia, reticulocytes, and microcytosis, was analyzed by optical microscopy at 1000×. The percentage of schistocytes and echinocytes in the peripheral blood of each experimental group was calculated by analyzing 10 fields/blood smear (*n* = 4)/each experimental group. The results were expressed as the mean ± SEM of the percentages of schistocytes and echinocytes.

#### 5.12.3. Free Hemoglobin

Blood samples were diluted with saline solution (1/100) and finally centrifuged at 1500 rpm for 10 min. Free hemoglobin was measured in the plasma fractions by reading the absorbance in an automated plate spectrophotometer at 540 nm.

### 5.13. Data Analysis

Data are presented as mean ± SEM. Statistical analysis was performed using Graph Pad Prism Software 5.0 (San Diego, CA, USA). ANOVA was used to calculate differences between groups and Tukey’s multiple comparisons test was used as posteriori. Survival of mice was analyzed by using Kaplan-Meier curves and Log-rank test analysis.

## Data Availability

Data are available upon request, please contact the contributing authors.
